# Robotic Spot Spraying of Harrisia Cactus (*Harrisia martinii*) in Grazing Pastures of the Australian Rangelands

**DOI:** 10.3390/plants10102054

**Published:** 2021-09-29

**Authors:** Brendan Calvert, Alex Olsen, James Whinney, Mostafa Rahimi Azghadi

**Affiliations:** 1 Bebegu Yumba Campus, College of Science and Engineering, James Cook University, Townsville, QLD 4811, Australia; james.whinney@jcu.edu.au (J.W.); mostafa.rahimiazghadi@jcu.edu.au (M.R.A.); 2 AutoWeed, Townsville, QLD 4814, Australia; alex@autoweed.com.au

**Keywords:** *Harrisia martinii*, Australian rangelands, robotic spot spraying, deep learning

## Abstract

Harrisia cactus, *Harrisia martinii*, is a serious weed affecting hundreds of thousands of hectares of native pasture in the Australian rangelands. Despite the landmark success of past biological control agents for the invasive weed and significant investment in its eradication by the Queensland Government (roughly $156M since 1960), it still takes hold in the cooler rangeland environments of northern New South Wales and southern Queensland. In the past decade, landholders with large infestations in these locations have spent approximately $20,000 to $30,000 per annum on herbicide control measures to reduce the impact of the weed on their grazing operations. Current chemical control requires manual hand spot spraying with high quantities of herbicide for foliar application. These methods are labour intensive and costly, and in some cases inhibit landholders from performing control at all. Robotic spot spraying offers a potential solution to these issues, but existing solutions are not suitable for the rangeland environment. This work presents the methods and results of an in situ field trial of a novel robotic spot spraying solution, *AutoWeed*, for treating harrisia cactus that (1) more than halves the operation time, (2) can reduce herbicide usage by up to 54% and (3) can reduce the cost of herbicide by up to $18.15 per ha compared to the existing hand spraying approach. The AutoWeed spot spraying system used the MobileNetV2 deep learning architecture to perform real time spot spraying of harrisia cactus with 97.2% average recall accuracy and weed knockdown efficacy of up to 96%. Experimental trials showed that the AutoWeed spot sprayer achieved the same level of knockdown of harrisia cactus as traditional hand spraying in low, medium and high density infestations. This work represents a significant step forward for spot spraying of weeds in the Australian rangelands that will reduce labour and herbicide costs for landholders as the technology sees more uptake in the future.

## 1. Introduction

### 1.1. Harrisia Cactus

Harrisia cactus (*Harrisia martinii*) is a perennial cactus native to Argentina and Paraguay that was introduced to Queensland, Australia in the 1890s [1]. It has since become a major weed of the Australian rangelands as it forms dense impenetrable thickets that compete with native pastures for nutrients and inhibits the movement of livestock [2]. With these severe impacts on grazing, the weed has consequently been a significant economic burden in Queensland for decades. Bradshaw et al. estimated that harrisia cactus has cost the Queensland economy $156M in weed management costs and lost production since 1960—the second most of any invasive species in that timespan [3].

**Figure 1:**
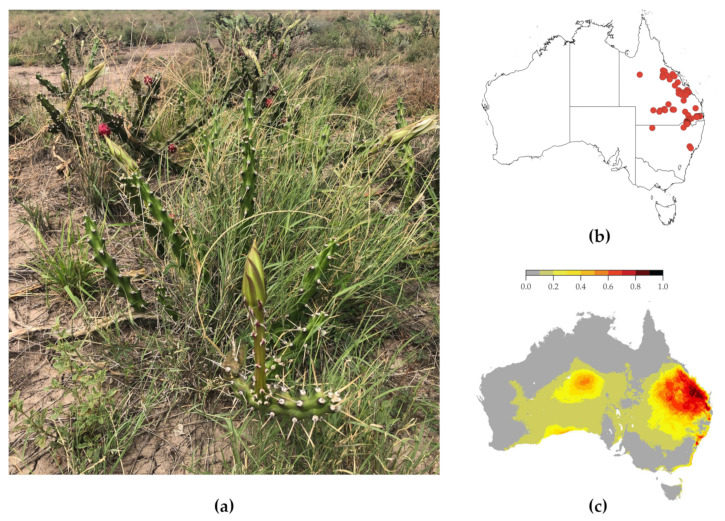
(**a**) Flowering plants of harrisia cactus in grazing pastures in northern New South Wales. (**b**) Current distribution of harrisia cactus in Australia from the Atlas of Living Australia data occurrence records [9]. (**c**) The current estimated percentage of suitable habitat for harrisia cactus in Australia [7].

Harrisia cactus is identifiable by its green fleshy-jointed stems that form tangled mats about 50 cm tall [4]. The plant also has large white funnel-shaped flowers and round bright red fruits with scattered bumps, hairs and spines (as pictured in Figure 1a). These strong features make the plant distinctly visible in diverse rangeland pastures with varied neighbouring flora.

The weed prefers habitats with deep clay soils and thrives in summer rainfall areas receiving 500–1300 mm annual rainfall [5]. In these suitable conditions, the rangeland weed outcompetes native pastures with infestations of up to 140,000 plants per ha and 80–90% ground cover [6]. The current occurrence records from the Atlas of Living Australia (Figure 1b) reveals the infestations of harrisia cactus spreading to northern Queensland and southern New South Wales. However, Duursma et al. [7] and Weed Futures Australia [8] estimate that much of the eastern coast of Australia provides a suitable habitat for harrisia cactus infestations (Figure 1c). This emphasises the need for advanced control mechanisms of this weed to prevent its wider spread.

### 1.2. Control Methods

Effective methods of control for harrisia cactus include physical removal, biological control, and herbicide control. Physical removal is time consuming and requires total removal of the weed, as severed portions can take root to develop new clusters. This is especially difficult for harrisia cactus due to its deep underground tuberous root system. As such, an integrated approach combining physical, biological and chemical methods is recommended [4].

A biological control program was undertaken in Queensland from 1973 to 1995 [5]. This effort was a significant investment from the Queensland Government with expenditure on the on-ground control of harrisia cactus in 1976 totaling $618,000 [10]. Widespread collection and redistribution of the most successful agent, the cactus mealybug (*Hypogeococcus festerianus*), took place throughout the 1980s, including into infestations in border areas of southern Queensland and northern NSW. The biological control program was regarded as an outstanding success, achieving and maintaining low levels of cactus infestation in northern and central Queensland where biological control alone is sufficient. However, in southern Queensland and adjacent New South Wales, where temperatures are cooler, the cactus mealybug develops more slowly and control is slow and less effective. In these areas, chemical control is preferred for more rapid control [5].

Chemical control has proven to be an effective control measure with hand-gun foliar application of registered herbicides being the most commonly used approach [4]. However, this is a time consuming and costly process due to: (1) the labour requirement to manually identify and spot spray target plants, (2) the high cost and quantity of herbicide to achieve control via foliar application, and (3) the weed’s deep rooted system requiring follow up application. In parts of southern Queensland and northern New South Wales, where biological control has faltered, landholders are spending $20,000 to $30,000 per year for chemical control costs [11]. As such, despite its effectiveness, the costs of chemical control have inhibited its usage by landholders in such areas with large infestations.

A potential solution to this problem is robotic spot spraying, which can potentially deliver faster, more reliable and consistent applications of herbicide to maximise the effectiveness of control while reducing the costs of labour and herbicide.

### 1.3. Robotic Spot Spraying

Robotic spot sprayers have been used increasingly in Australian agriculture in the past decade with commercial products such as the WEEDit [12] and WeedSeeker [13] delivering economic savings on weed management costs for the broadacre cropping industry [14]. These two robotic spot spray solutions exploit the spectral absorption properties of plants as the basis for detection and spot spraying. They emit a laser light onto a field of view within which any living plant reacts photosynthetically and its response is sensed by the detection unit. Any and all sensed plants are immediately treated with herbicide as the boom sprayer traverses the crop. These systems deliver chemical savings by applying herbicide only where it is needed. However, they do not differentiate between crop and weed, which limits their use to spraying of weeds and volunteer crops in fallow situations (i.e., *green-on-brown*). The industry need and research focus for *green-on-green* robotic spot sprayers that can distinguish between weeds and crops (or pastures) is palpable due to its wide ranging applications. Consequently, this space has seen rapid research and development in recent years [15].

Except for a handful of works [16,17,18], the majority of research and development has focused on broadacre crop application with robotic control of weeds in rangeland pastures, like harrisia cactus, remaining an underdeveloped field. Robotic spot spraying of harrisia cactus requires a green-on-green solution to distinguish the cactus from native pasture with the added difficulties of high variability of off-target weed species, native flora, lighting conditions and less common environmental landmark features in the rangeland environment. Past work by the authors showed that in situ detection of harrisia cactus with artificial intelligence could achieve an average classification accuracy of up to 98% [17]. This work showed the promise of a green-on-green approach, however it lacked a comprehensive trial of such technology to examine its efficacy in comparison with existing control methods. A robotic spot sprayer for the Australian rangelands requires different specifications than the current commercial offerings in broadacre cropping. A rangeland system must have a narrower boom width, with a robust design that can operate in the difficult rangeland terrains.

### 1.4. Overview

This work presents a novel robotic spot spraying system for treating harrisia cactus (and other weeds of the Australian rangelands) amongst native pastures. Section 2 documents the design methodology for the robotic system and the detection methods. Section 2 also provides an overview of the experimental design for evaluating the spot spraying approach versus traditional hand spraying in terms of time, costs savings and weed knockdown efficacy. Section 3 reports on the results from the detection and spot spraying experiments and discusses the implications of this work for the industry. Section 4 concludes with a look toward future challenges for robotic spot spraying solutions in rangeland environments. Furthermore, a supplementary video that summarises the aims, methods and results of this work has been provided in the Supplementary Materials.

## 2. Materials and Methods

### 2.1. Trial Site

**Figure 2:**
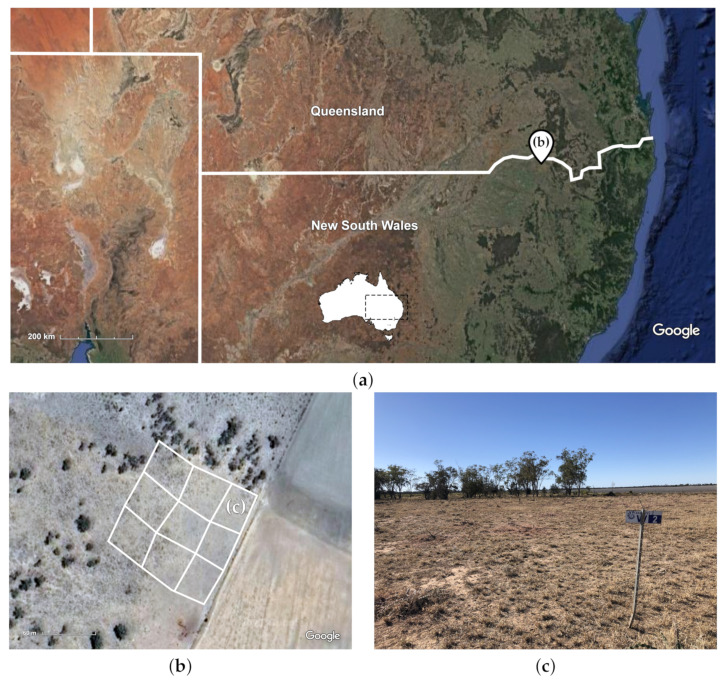
(**a**) A map of the trial site at Warakirri Cropping’s Willaroo farm located on the QLD-NSW border (Imagery ©2021 TerraMetrics, Map data ©2021 Google). (**b**) Nine trial plots with varying levels of harrisia cactus infestation (Imagery ©2021 CNES/Airbus, Maxar Technologies, Map data ©2021). (**c**) An up close look at a low density trial plot.

Willaroo farm, located 22 km south west of the Queensland border town of Goondiwindi, was chosen as the trial location for this work with its sprawling rangeland pastures, 600 mm of average annual rainfall, and dense infestations of harrisia cactus (Figure 2). It is owned and operated by Warakirri Cropping, whose production focus at Willaroo is grain crops of wheat, barley and sorghum. However, their lands contain over 10,000 ha of pasture with dense infestations of harrisia cactus. The staff at Willaroo perform manual hand spraying of harrisia cactus infestations while traversing the rangeland paddocks with a John Deere Gator UTV. This location provides an ideal trial site to evaluate the efficacy of a new spot spraying approach by comparing the labour requirement, herbicide costs, and weed knockdown rates to the existing hand spraying control method being used. A paddock with varying levels of harrisia cactus thickets was divided into nine 0.25 ha trial plots to conduct individual spray assessments (Figure 2).

### 2.2. AutoWeed Detection Unit

**Figure 3:**
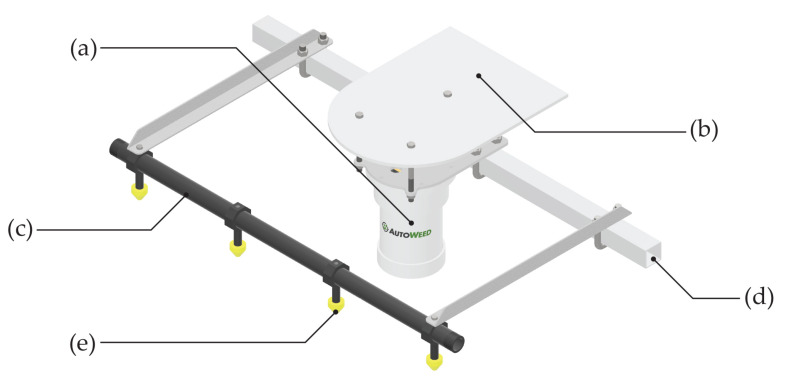
AutoWeed’s proprietary weed detection and spraying boom mount design for a 1 metre boom section, including: (**a**) the AutoWeed detection unit, (**b**) a protective sun shade, (**c**) a 1” wet boom, (**d**) a 40 × 40 mm steel hollow section frame, and (**e**) TeeJet solenoids and nozzle body adaptors.

This project utilises proprietary weed detection and spraying units developed by AutoWeed (as shown in Figure 3). These units were purpose built for use in the rangeland environment and can be retrofitted to existing agricultural vehicles to suit a specific application. Each unit comprises a machine vision camera, an NVIDIA Jetson embedded GPU processor, and a custom solenoid sprayer board that can individually control up to four solenoids per camera. The design is enclosed in a robust PVC housing attached to a mounting plate to protect the internals from the harsh environment. The units can be mounted to any 40 mm or 50 mm steel hollow section frame and is compatible with TeeJet boom and spray nozzle components. For weed detection, the units utilise the latest deep learning based image classification models that are custom trained to weeds of interest using large labelled image datasets following the methodology of [18].

### 2.3. Deep Learning

Deep convolutional neural networks (CNN) are the gold standard in image classification. They are capable of accurately extracting and learning unique image features from large labelled image datasets. The ImageNet Large Scale Visual Recognition Challenge (ILSVRC), established in 2010 [19], has largely been dominated by CNNs since 2012 when a CNN far outperformed other machine learning methods of the time [20]. Since then, CNN variants have seen widespread adoption across industry, while researchers continue to incrementally improve CNN architectural design to be more and more accurate. The introduction of simple tools and coding libraries such as Google’s TensorFlow and Facebook’s PyTorch has made machine learning more accessable to researchers.

Our past work presented *DeepWeeds*, the world’s first large image dataset of weeds from the Australian rangelands, upon which a ResNet-50 [21] classifier achieved 95.7% classification accuracy [18,22]. A follow up case study showed that up to 98.1% accuracy could be achieved using the same deep learning based approach to detect harrisia cactus in situ [17]. To implement this detection in real-time, [16] used the lightweight MobileNetV2 network [23] and NVIDIA’s TensorRT acceleration library to reduce latency and allow spot spraying to be performed. These methodologies are combined in this work aboard the AutoWeed units to detect harrisia cactus for real-time spot spraying.

The same deep learning training regime from [16,17,18] is used here by implementing transfer learning and image augmentations to reduce overfit on the new harrisia cactus datasets. Transfer learning uses a pre-trained model from a much larger and more general dataset (i.e., ImageNet) to transfer its wide ranging feature set to a new learning domain (i.e., harrisia cactus in pastures) [24]. This effectively improves results on small scale image datasets where feature variability is low. Image augmentations provide a similar function to expand the feature set of a limited domain. This artificial variation reduces the risk of model overfit and results in a more generalised model. Previously, our work used the pre-trained model ResNet-50 to achieve the best results [17,18]. It utilises residual functions to gain accuracy from increased architectural depth [21]. However, ResNet50 is a large network and can be too slow for real-time application. Hence to improve the speed of processing for the spot sprayer, a pre-trained lightweight MobileNetV2 [23] model is trained and deployed as in [16].

### 2.4. Vehicle and Spray Boom Design

For the purposes of this project, Warakirri Cropping’s Willaroo operation supplied a John Deere Gator UTV to serve as the base vehicle for a robotic spot spraying system. To prepare the UTV for spray trials, a custom three metre boom was developed and installed onto the UTV by SprayerBarn Moree.

The boom design uses a 300 L Goldacres T3 tank with a 20 L/min, 20 bar diaphragm pump to deliver chemical application rates of over 1000 L/ha. The spray jets for this system include TeeJet eChemsaver electric solenoid shutoff valves with TeeJet FloodJet nozzles for high volume herbicide delivery. The boom covers a three metre spraying swath with four AutoWeed detection units, each covering a field of view of 750 mm and controlling three solenoid spray jets per unit. The system also includes a manual handgun on the spray tank to allow the same vehicle to be used for manual hand spraying.

**Figure 4:**
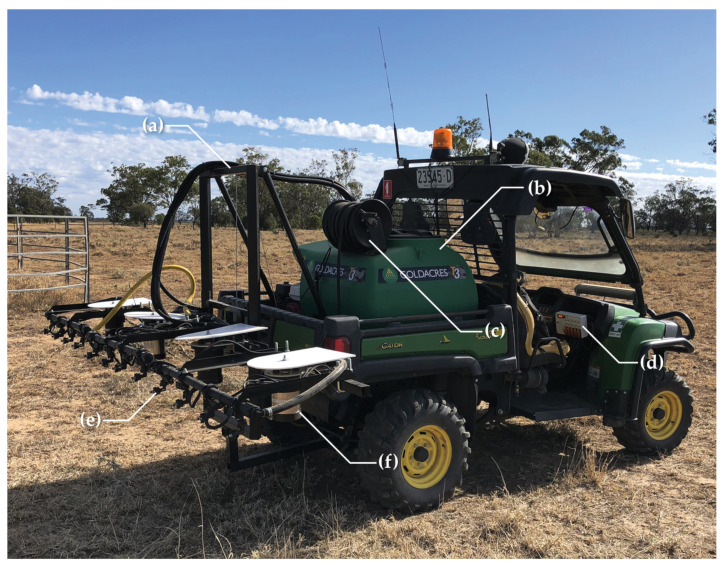
A John Deere Gator UTV with an installed three metre AutoWeed spot spraying system, including: (**a**) a winch and frame for electronic height adjustment, (**b**) a 300 L Goldacres T3 tank and a 20 L/min diaphragm pump with a Honda GX120 engine and manual pressure regulator, (**c**) a spray handgun and reel for manual spraying, (**d**) an AutoWeed control box to monitor unit operation, (**e**) a 1” wet boom with twelve TeeJet solenoids, nozzle bodies and Turbo FloodJet spray nozzles, and (**f**) four AutoWeed detection units.

To enable control of the detector units, a control box was installed into the cabin of the UTV (as pictured in Figure 4). The control box allows the operator of the vehicle to power the units on and off, observe the status of each detection unit during operation and perform manual override of the solenoids to purge chemical from the spray line after usage. While performing spraying, status lights on the control box will illuminate indicating a weed has been spotted and sprayed.

### 2.5. Dataset Collection and Labelling

To calibrate the AutoWeed detection units to target harrisia cactus, a new learning model must be trained and installed on the units. To achieve this, a large dataset is required that is comprised of a mix of target and non-target images from harrisia cactus infested pastures. The larger and more variable the dataset, the stronger the learning algorithm will be. Ideally the dataset should capture the target environment in different weather situations, lighting levels, times of day, and with different stages of growth and variety of flora. This will help to create a diverse dataset to encourage the learning algorithm to learn features that are specific to harrisia cactus.

The developed three metre spot spraying system can be used to rapidly collect a large image dataset in the target environment where spraying will be performed. The AutoWeed detection models were set to data collection mode while the vehicle was driven over the rangeland pastures collecting target and non-target images. All images are saved to local storage on the detection units. The images can then be labelled as to whether they contain target harrisia cactus or not using AutoWeed’s proprietary image labelling software, which allows for labelling up to 2000 images per hour.

### 2.6. Chemical Mixture and Nozzle Selection

North West Local Land Services completed an extensive trial of herbicide mixture effectiveness for harrisia cactus control in 2017 [25]. This work showed that a variety of chemical mixtures achieved effective knockdown of harrisia cactus stools at varying price points. Perhaps the most cost effective approach that achieved acceptable knockdown were combinations of Grazon Extra, metsulfuron-methyl and a wetting agent; which achieved 99% efficacy with a chemical cost of $8–9 per 100 L of chemical mixture. This is the chemical mixture of choice for controlling harrisia cactus at Warakirri Cropping’s Willaroo operation. The specific products for this trial were prepared by the staff at Willaroo station and are outlined in Table 1. The chemical cost for this mixture totals $12.97/100 L.

This same chemical mixture was used for all sites in the trial. However, low and high volume applications were applied to gauge the weed kill efficacy with different levels of chemical coverage on the weed. Effective herbicide control of harrisia cactus requires 1000 to 1500 L/ha foliar application until the cactus is thoroughly wet [4]. A low volume spray application of 927 L/ha can be performed using the AutoWeed spot sprayer by installing four TeeJet FloodJet TF-VS10 nozzles and eight TF-VP4 nozzles while travelling at 10 km/hr with a line pressure of 2 bar. In contrast, a high volume spray application of 1548 L/ha is performed using twelve TF-VS10 nozzles while travelling at 10 km/hr with a line pressure of 2 bar.

### 2.7. Experimental Methodology

**Figure 5:**
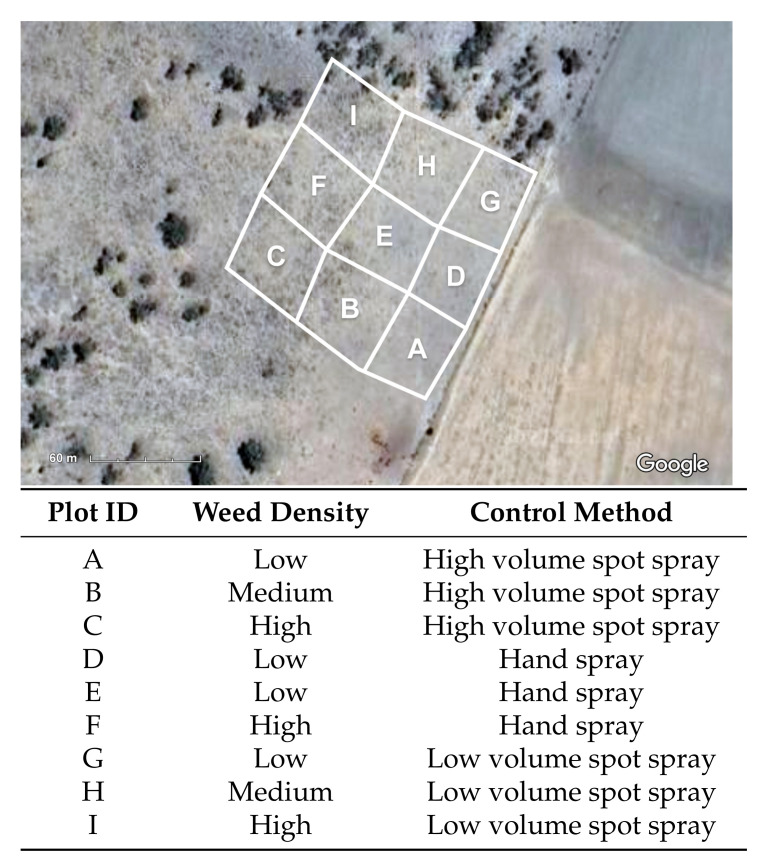
A map of the nine designated 0.25 ha trial plots for evaluating spot spraying vs. traditional hand spraying (**top**) and their associated weed density and control method for the trials (**bottom**). (Imagery ©2021 CNES/Airbus, Maxar Technologies, Map data ©2021).

Nine individual 0.25 ha spray plots were mapped out to allow comparison of different chemical application treatments. The GPS locations of the area boundaries for each 0.25 ha spray plot are mapped in Figure 5. The nine areas are categorised as having low, medium or high density infestations of harrisia cactus based on the following criteria, where *n* is the approximate number of individual harrisia cactus plants in the 0.25 ha plot area:

Low: 0 < *n* < 100
Medium: 100 < *n* < 200
High: *n* > 300

Three different chemical treatments were applied across the different plots to compare hand spraying against low and high volume robotic spot sprays. For each spray trial, data was recorded for the time taken, herbicide used, and weed knockdown achieved across the different levels of weed infestations. The three chemical treatments are further described below:

Hand spray: Hand spraying with a handgun nozzle while traversing the plot with the UTV at varying speeds and manually identifying individual plants.
Low volume spot spray: AutoWeed robotic spot spraying with a low volume (927 L/ha) application rate while traveling over the plot at <=10 km/hr in straight line transects.
High volume spot spray: AutoWeed robotic spot spraying with a high volume (1547 L/ha) application rate while traveling over the plot at <=10 km/hr in straight line transects.

The speed of the vehicle during the trial relies on the terrain, where the terrain was too rough, slower speeds were used. The sites were visually inspected after herbicide application was completed by observing the distribution of red dye in the chemical to imperically evaluate foliar coverage. A further inspection was conducted three months after the spray trials to determine kill rates in each of the sites. A thorough analysis of the results was then performed to determine the overall performance of each method. It is important to note that the spray trials were conducted during drought conditions, when the biological control methods are least effective.

## 3. Results and Discussion

### 3.1. Dataset Collection

**Figure 6:**
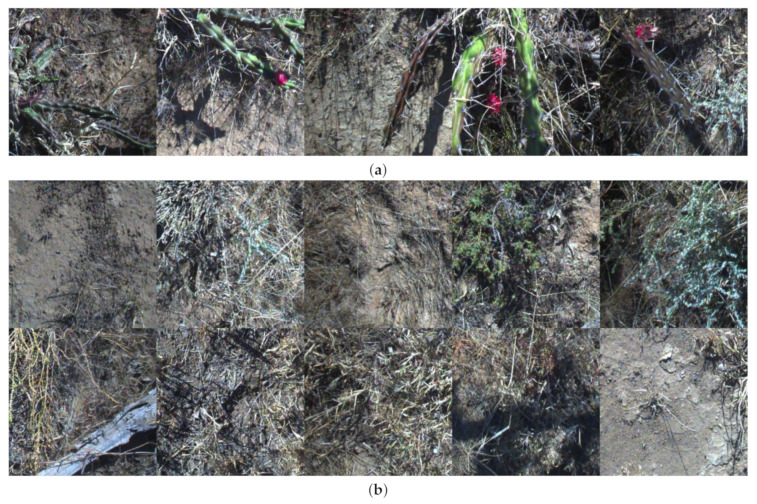
Random sampling of (**a**) harrisia cactus and (**b**) negative images from the collected dataset. Note there is an inherent imbalance in this dataset with approximately six negative images for every positive harrisia cactus image matching the real world distribution seen in the trial location.

More than 100,000 images were collected during the dataset collection phase of the trial in June 2020. These images were sub-sampled by a factor of two to avoid pseudoreplication of samples caused by frame overlap between successive frames. The total count of images was 58,153 images which were then labelled by human experts. AutoWeed’s proprietary labelling software was used to label the images. Target images were labelled as “harrisia cactus” and non-target images were labelled as “negative”. The positive class (illustrated by random samples in Figure 6a) includes any image that contains harrisia cactus. Cactus plants during collection varied between dry and good health with many plants bearing fruits. Meanwhile, the negative class (illustrated by random samples in Figure 6b) includes any image that does not contain harrisia cactus. This makes for a highly variable negative class including pasture, dirt and other plant life.

The final class distribution for the dataset included 7782 positive harrisia cactus images and 50,371 negative images (see Table 2). This class imbalance matches the natural weed density of the affected pastures at Willaroo farm.

### 3.2. Deep Learning Training

Following the labelling process, the 58,153 image dataset was randomly split into 80% and 20% subsets of training and validation, where 80% of the labels were set aside for training and 20% of the labels were set aside for model validation. The class distribution between training and validation subsets was stratified such that the ratio of the negative class to the harrisia cactus class was maintained at 6.47 across all subsets (see Table 2).

To allow for benchmarking of accuracy metrics for researchers, machine learning datasets are usually split three ways with training and validation subsets, and a test subset that is heldout to test the model. We have foregone this data splitting approach here in order to train models on the most data possible. Furthermore, the test set for this work is the real-time in situ data when evaluating spraying performance in the field.

**Figure 7:**
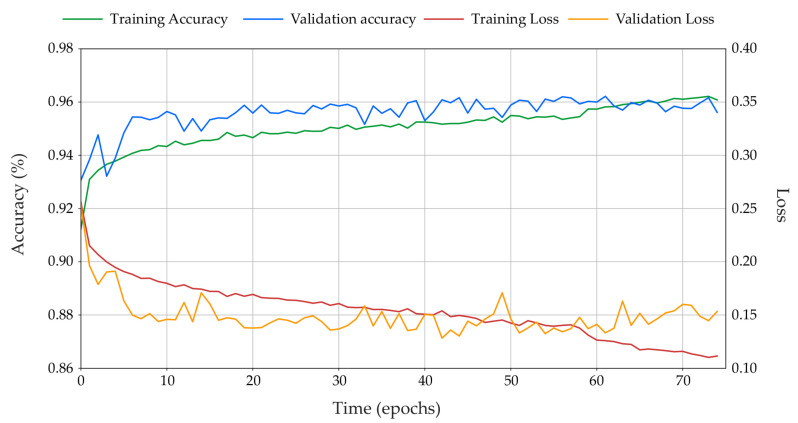
Training and validation accuracy versus epoch (left axis) and training and validation loss versus epoch (right axis) during the training process.

The TensorFlow machine learning backend was used together with the Python-based high level API Keras, to train a MobileNetV2 architecture following the training methodology of [16,17,18]. The result of the training process is illustrated with time series plots of training and validation metrics versus successive epochs in Figure 7.

**Figure 8:**
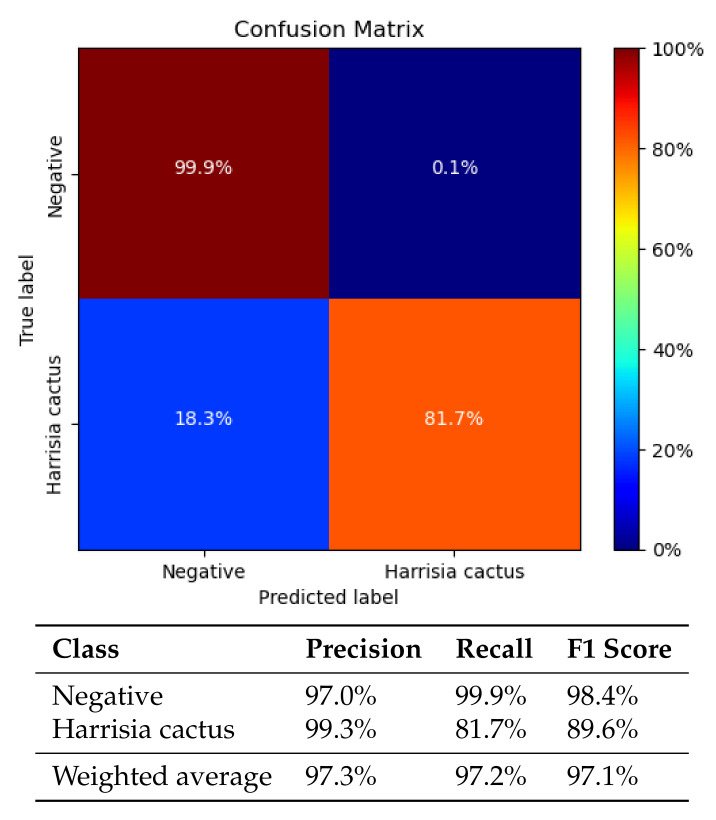
The confusion matrix (**top**) and the reported precision, recall and f1-score metrics (**bottom**) evaluating the performance of the MobileNetV2 harrisia cactus classifier on the validation subset.

As the training accuracy increases, so too does the validation subset accuracy, plateauing with a similar average classification accuracy of 96%. The model begins to overfit the dataset after 57 epochs, when the validation loss begins to increase as the training loss continues decreasing. Early stopping was used to halt training when the validation loss failed to decrease after 32 successive epochs. The ideal MobileNetV2 model for field implementation was arrived at after 42 epochs with a validation loss of 0.128 and an average classification accuracy of 96.1% on the validation subset. This model was saved in order to be deployed on the AutoWeed detection units for subsequent spraying trials. However, the metric of average validation accuracy does not give a clear indication of model performance. To capture how well the model performs on the two individual classes we must review individual class classification results and the confusion matrix (see Figure 8).

### 3.3. Classification Results

The MobileNetV2 model performs well at classifying negatives with a true negative rate of 99.9%, while only having 0.1% false positive predictions (see Figure 8). This incredibly low false positive rate is a great outcome for a spot sprayer in rangeland pastures, effectively limiting the amount of off-target damage to native pastures from misapplied herbicide. This is also important as the spot sprayer would most often encounter low infestation levels across much of the rangelands except for highly dense areas, which are less common.

However, harissia cactus classification accuracy is much lower at a rate of 82% (see Figure 8), where the remaining 18% represents false negative predictions (missed images containing weeds). These results are lower than expected and perhaps are due to the unbalanced nature of the dataset biasing the model toward performing better on negative class prediction at the cost of positive class prediction. However, the true measure of the predictive power of the model will be seen with the classification accuracy on unseen data in the field. The 82% accurate image classification rate is likely to improve when deploying the model in the field since each plant target will be captured multiple times due to the 16 fps frame rate achieved with the MobileNetV2 model.

**Figure 9:**
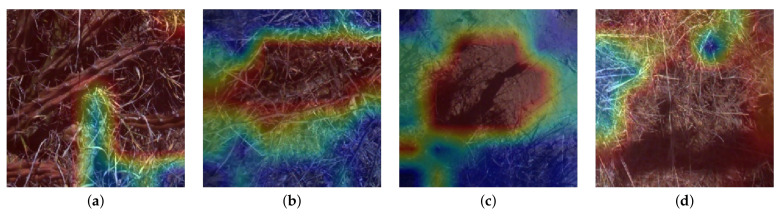
Class activation heatmaps that show where in an image the MobileNetV2 classifier finds features that most represent the negative class (blue) and the harrisia cactus class (red). (**a**) Most confident true positive prediction: 100% harrisia cactus, (**b**) Least confident true positive prediction: 50.3% harrisia cactus, (**c**) Random false positive prediction: 89.1% harrisia cactus, (**d**) Random false negative prediction: 99.5% negative.

Further analysis of the image classifier is useful to help understand what image features the model has been trained to recognise as harrisia cactus. For this we generate heatmaps of images that have been processed by the model (see Figure 9) using a method known as class activation maps, which allows for visual confirmation that the model has learned the desired target, while exposing what might be potentially confusing the model [26].

The most confident true positive in Figure 9a shows the the model is correctly focusing on features specific to harrisia cactus while ignoring features that represent native pastures. Figure 9b shows the least confident true positive, where the confusion appears to be from a dead tube of harrisia cactus. Figure 9c reveals the cause of a false positive to be a shadow from a harrisia cactus branch fooling the model. This issue represents realistic limitations of the model that the class activation maps were able to highlight. This is, however, of minor impact due to the low occurrence of such phenomena and should be considered for inclusion in future models in locations where these examples are more common. The false negative from Figure 9d is caused by a small bit of harrisia cactus in the lower right corner which may be too small a feature for the model to recognise. Fortunately, with a high frame rate, successive frames following such an image would present the cactus more central in the field of view for positive detection.

### 3.4. Spray Trial Results

The aforementioned MobileNetV2 classifier was converted into a TensorRT inference engine and deployed on the AutoWeed detection units for in-field spraying trials in June 2020. Spraying was conducted across the nine plots following the control methodologies outlined in Figure 5. The results for these field trials are summarised in Table 3. Some of the results (time taken, chemical used and estimated weed count) were immediately recorded following the spray trials in June 2020, whereas other metrics (weeds killed and weeds missed) were estimated by Willaroo staff following a three month follow up inspection of the trial sites. These results will be further analysed to compare the three control methods in terms of weed knockdown, labour requirements and herbicide usage. It is worth noting that due to limited sample size (i.e., only three replications for each spray method in three different infestation levels) the results are not statistically significant at the 95% confidence threshold. Where possible, we have quoted the standard deviation of averaged results.

#### 3.4.1. Weed Knockdown

**Figure 10:**
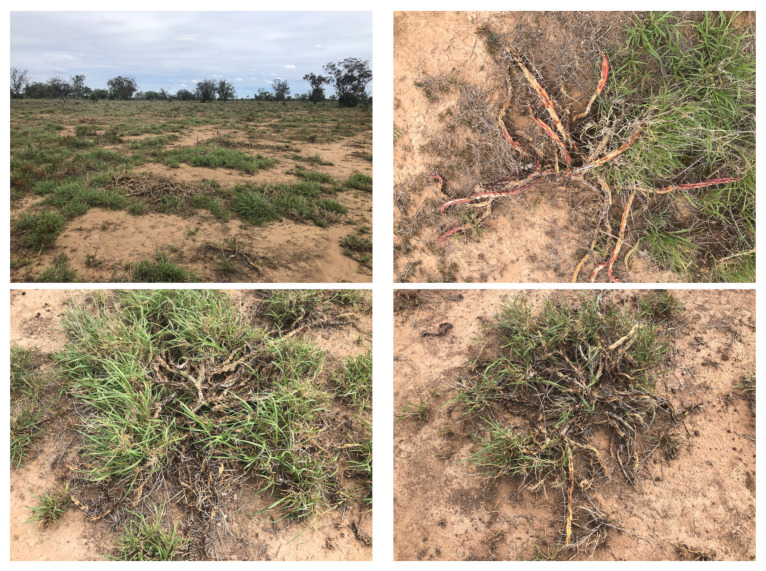
Examples of weed knockdown across the trial plots following the three month follow up inspection after spraying.

We analysed average weed knockdown (or kill rate) for each control method across low, medium and high density infestations (see Table 4). Hand spraying achieved the highest level of average weed knockdown with 98.3% ± 1.5% likely due to excess coverage; while AutoWeed robotic spot spraying achieved comparable knockdown at 95.0% ± 1.7% and 96.3% ± 0.6% for low and high volume applications, respectively. The high volume application appeared to have slightly better weed knockdown than low volume spraying, which is to be expected due to the larger herbicide coverage. However, due to the limited sample size of the trials, these results are not statistically significant to the 95% confidence threshold. Photographs of the three month follow up inspection at the nine trial sites were taken showing examples of the strong weed knockdown achieved by each control method (see Figure 10). Pasture recovery at these sites was ample following strong rains in the region between the date of the trials and the follow up.

Similarly, the hand spraying approach achieved slightly better spraying accuracy with fewer missed targets compared with the robotic spot spraying applications (see Table 5). This is likely due to the increased time taken by the human operator to scout and spray weed targets for the 0.25 ha plot areas. However, it would be expected that human performance would vary over prolonged time periods, while the robotic spot sprayer would maintain its level of performance assuming it was set up accordingly each time. It is interesting to see such a high in situ accuracy from the robotic spot sprayer despite the theoretical model accuracy of 81.7% for harrisia cactus. We believe this is due to a combination of the speed of the inference algorithm and the speed of the vehicle, which on average was approximately 7 km/h. This presented the model with multiple attempts at classifying the weed images correctly as the speed is slow enough to capture multiple images of the same target.

**Figure 11:**
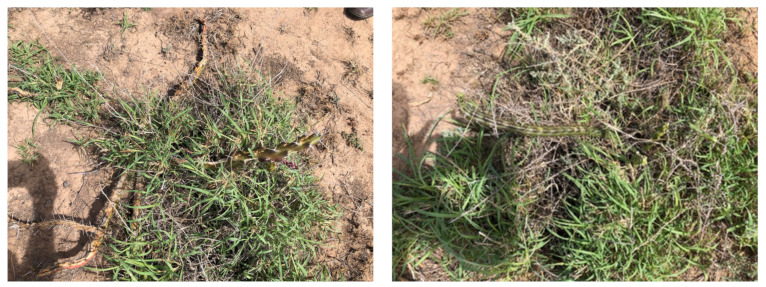
Examples of missed harrisia cactus plants from the nine spray trials that were evident due to their large growth size at the time of the three month follow up inspection.

**Figure 12:**
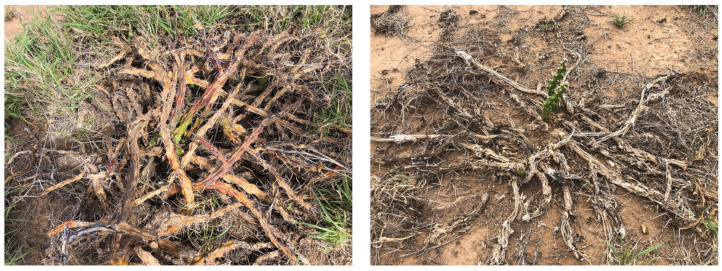
Examples of harrisia cactus plant regrowth across the nine trial plots identified during the three month follow up inspection.

Photographs from the three month follow up inspection by Warakirri Cropping reveal instances of missed targets (see Figure 11) and regrowth of harrisia cactus plants that were successfully sprayed (see Figure 12). Harrisia cactus regrowth is a common occurrence after herbicide control due to the deep root system of the plant. The high volume application rate is preferred to reduce the chance of regrowth but more testing could help develop methods for improved coverage at lower volumes.

#### 3.4.2. Herbicide Usage

In all spray trials and for all weed density levels, the amount of herbicide mixture used during hand spraying exceeded the amount used in robotic spot spraying (see Table 6). This is an expected result since hand gun nozzles output chemical at a faster rate and human operators are likely to spend a longer time spraying target plants compared with the robotic spot sprayer which passes over at a constant speed.

Due to the small-scale trial statistical significance is hard to quantify, so herbicide usage was normalised against the highest applied amount for each respective density to better highlight their comparative differences (see Table 6). The only exception to this were the medium density results, due to the hand spray trials not containing a true medium density infestation. For this purpose low density areas with greater than 100 targets were analysed against the medium density areas. The results from Table 6 consistently show a clear reduction in herbicide for low and high density infestations when boom spraying. This is also true for medium density infestations, although not immediately obvious, as they achieved comparable numbers against a low density area for hand spraying that had lower targets but comparable herbicide usage.

An interesting result to note is that as the infestation level increases, lower savings are observed. We believe this is because overuse of herbicide in low density areas is easier when hand spraying singular targets due to the human element. Looking at the high density area results, the comparison between hand spraying and high volume boom spraying show a 14% reduction in herbicide when using high volume boom spraying which is a lower reduction than for low density areas. Low volume boom spraying shows a 48% reduction in herbicide usage in high density areas and seems to counter the observation. However, this high reduction is misleading, as this area contains roughly 100 fewer weeds than the other two high density areas and would lead to less herbicide usage. The human element can yield unpredictable and inconsistent results so exact comparisons are hard to make. It is however clear from these results that boom spraying with the robotic spot sprayer yields a significant decrease in herbicide usage.

The reduction in herbicide afforded by robotic spot spraying comes with a quantifiable reduction in weed management costs due to savings made on chemical usage. Table 7 shows that hand spraying during these trials was the most expensive control method at $49.26 ± $22.60 per ha. This figure exceeded both boom spraying approaches with the low and high volume applications costing $31.11 ± $9.35 per ha and $38.89 ± $23.76 per ha, respectively.

#### 3.4.3. Labour Requirement

Finally, we assess the labour requirement of each control method by comparing the time taken to carry out each spray trial for all three control methods across low, medium and high density weed levels (see Table 8). Total time savings from 32% to 73% were observed when using AutoWeed robotic spot spraying rather than traditional hand spraying. This significant time saving makes sense due to the labourious nature of hand spraying and the relative ease of boom spraying at a constant speed while traversing the paddock. These time savings will constitute a further cost saving in labour.

## 4. Conclusions

This work presents a novel solution for robotic spot spraying of harrisia cactus in rangeland pastures. This new solution is a threefold improvement on traditional hand spraying by: (1) reducing the time of operation and associated labour costs by 32–73%; (2) using between 14 and 54% less chemical than hand spraying and consequently saving between $10.37–$18.15 per ha on herbicide costs; while (3) achieving similar levels of weed knockdown and spraying accuracy.

Despite these strong results, there are still some limitations with the robotic spot spraying solution; which will likely improve as the technology matures. Firstly, this system cannot travel faster than 10 km/hr due to maximisation of the processor being utilised. Commercial systems that require faster operating speeds can utilise higher power processors in the future. There were also weak points identified in the deep learning model, where outlying edge cases of shadowed targets and unhealthy plants were able to confuse the detection model. This can be further improved by feeding these edge case samples back into the model for another round of training. Finally, the spray activation mechanism fails to account for ground speed and elevation, and so, when the terrain is uneven or when the vehicle speed drops below its calibrated constant speed, spraying accuracy is compromised. Future work will look at adding motion sensing equipment to track the vehicle’s motion in order to improve spray accuracy. Furthmore, trials were conducted during drought conditions, so trials during the wet season should be performed to test the year-round effectiveness to determine the best timing for herbicide control. Despite these limitations this work represents a strong step forward for robotic weed control in rangeland environments.

## Figures and Tables

**Table 1:** Chemical mixture used to control harrisia cactus throughout all spray trial experiments. Note that red dye was used to assess spray coverage for the trials, and its costs are not included in the cost analysis for the chemical mixture.

Product	Description	Volume (per 100 L Mixture)	Price (per 100 L Mixture)
Grazon Extra	Group I herbicide	300 mL	$10.49
Activator Surfactant	Wetting agent	300 mL	$2.08
Associate	Group B herbicide	5 g	$0.40
Red dye	Spray marker	75 mL	-

**Table 2:** Distribution of images by class within the collected dataset which is stratified for the training and validation subsets.

	Harrisia Cactus	Negatives	Total Images
Training	6226	40,296	46,522
Validation	1556	10,075	11,631
Total	7782	50,371	58,153

**Table 3:** Results comparing the AutoWeed robotic spot spraying solution to traditional hand spraying in terms of time of operation, herbicide usage and weed knockdown.

Plot ID	Weed Density	Control Method	Time Taken (mins)	Chemical Used (L)	Estimated Weed Count	Estimated Weeds Killed (%)	Estimated Weeds Missed (%)
A	Low	High volume spot spray	14	35	<100	96	4
B	Medium	High volume spot spray	15	65	>200	96	2
C	High	High volume spot spray	16	125	>400	97	0.75
D	Low	Hand spray	25	75	<100	100	0
E	Low	Hand spray	25	65	>100	97	3
F	High	Hand spray	48	148	>400	98	1.25
G	Low	Low volume spot spray	11	40	>100	93	3
H	Medium	Low volume spot spray	17	65	>200	96	2.5
I	High	Low volume spot spray	13	75	>300	96	0

**Table 4:** Average and standard deviation of weed knockdown results achieved for each control method across all infestation levels.

Control Method	Average Weed Knockdown (%)
High volume spot spray	96.3±0.6
Hand spray	98.3±1.5
Low volume spot spray	95.0±1.7

**Table 5:** Average weeds missed and spraying accuracy achieved for each control method across all infestation levels. Note that spraying accuracy is calculated as 100%—weeds missed (%).

Control Method	Average Missed (%)	Spraying Accuracy (%)
High volume spot spray	2.3±1.6	97.8±1.6
Hand spray	1.4±1.5	98.7±1.5
Low volume spot spray	1.8±1.6	98.2±1.6

**Table 6:** The quantity of herbicide used for each control method in each level of weed density, normalised against the hand spraying herbicide usage levels to measure the reduction in chemical usage of the spot spraying approaches. Note that with only one replication for each trial, no statistical significance or standard deviation could be reported.

Control Method	Weed Density	Herbicide Used (L)	Normalised Herbicide
High volume spot spray	Low	35	0.46
	Medium	65	1
	High	125	0.86
Hand spray	Low	75	1
	Low	65	1
	High	145	1
Low volume spot spray	Low	40	0.53
	Medium	65	1
	High	75	0.52

**Table 7:** Comparison of costs for each control method determined from the average herbicide usage (L/ha) for each control method across all weed density levels using the chemical mixture from Table 1.

Control Method	Usage (L/ha)	Cost ($/ha)
Boom spray high volume	300±183	38.89±23.76
Hand spray	380±174	49.26±22.60
Boom spray low volume	240±72	31.11±9.35

**Table 8:** The time taken for each control method to cover each weed density level, normalised against the time required for the hand spraying approach in order to quantify the time savings made possible by spot spraying. Note that with only one replication for each trial, no statistical significance or standard deviation could be reported.

Control Method	Weed Density	Time Taken (mins)	Normalised Time
High volume spot spray	Low	14	0.56
	Medium	15	0.6
	High	16	0.33
Hand spray	Low	25	1
	Low	25	1
	High	48	1
Low volume spot spray	Low	11	0.44
	Medium	17	0.68
	High	13	0.27

## Data Availability

Restrictions apply to the availability of these data. Data was obtained from AutoWeed Pty Ltd. and are available from the corresponding author with the permission of AutoWeed Pty Ltd.

## Supplementary Materials

The supplementary video to this work, “AutoWeed: Spot spraying harrisia cactus in rangeland pastures” is available online at https://www.youtube.com/watch?v=4wn007Rtqoc.
